# Molecular Identification and Toxin Analysis of *Alexandrium* spp. in the Beibu Gulf: First Report of Toxic *A. tamiyavanichii* in Chinese Coastal Waters

**DOI:** 10.3390/toxins13020161

**Published:** 2021-02-18

**Authors:** Yixiao Xu, Xilin He, Huiling Li, Teng Zhang, Fu Lei, Haifeng Gu, Donald M. Anderson

**Affiliations:** 1 Key Laboratory of Environment Change and Resources Use in Beibu Gulf, Ministry of Education, Nanning Normal University, Nanning 530001, China; hexilin18177140136@163.com (X.H.); huiling18307713906@163.com (H.L.); zhangteng910410@163.com (T.Z.); 2 Guangxi Laboratory on the Study of Coral Reefs in the South China Sea, Guangxi University, Nanning 530004, China; 3 Guangxi Key Laboratory of Marine Environmental Science, Beibu Gulf Marine Research Center, Guangxi Academy of Sciences, Nanning 530007, China; leifu197510@163.com; 4 Third Institute of Oceanography, Ministry of Natural Resources, Xiamen 361005, China; guhaifeng@tio.org.cn; 5 Biology Department, Woods Hole Oceanographic Institution, Woods Hole, MA 02543, USA; danderson@whoi.edu

**Keywords:** *Alexandrium tamiyavanichii*, paralytic shellfish poisoning, molecular identification, toxicity, harmful algal blooms, Beibu Gulf

## Abstract

The frequency of harmful algal blooms (HABs) has increased in China in recent years. Information about harmful dinoflagellates and paralytic shellfish toxins (PSTs) is still limited in China, especially in the Beibu Gulf, where PSTs in shellfish have exceeded food safety guidelines on multiple occasions. To explore the nature of the threat from PSTs in the region, eight *Alexandrium* strains were isolated from waters of the Beibu Gulf and examined using phylogenetic analyses of large subunit (LSU) rDNA, small subunit (SSU) rDNA, and internal transcribed spacer (ITS) sequences. Their toxin composition profiles were also determined using liquid chromatography-tandem mass spectrometry (LC-MS/MS). All eight strains clustered in the phylogenetic tree with *A. pseudogonyaulax*, *A. affine*, and *A. tamiyavanichii* from other locations, forming three well-resolved groups. The intraspecific genetic distances of the three *Alexandrium* species were significantly smaller than interspecific genetic distances for *Alexandrium* species. Beibu Gulf isolates were therefore classified as *A. pseudogonyaulax*, *A. affine*, and *A. tamiyavanichii*. No PSTs were identified in *A. pseudogonyaulax*, but low levels of gonyautoxins (GTXs) 1 to 5, and saxitoxin (STX) were detected in *A. tamiyavanichii* (a total of 4.60 fmol/cell). The extremely low level of toxicity is inconsistent with PST detection above regulatory levels on multiple occasions within the Beibu Gulf, suggesting that higher toxicity strains may occur in those waters, but were unsampled. Other explanations including biotransformation of PSTs in shellfish and the presence of other PST-producing algae are also suggested. Understanding the toxicity and phylogeny of *Alexandrium* species provides foundational data for the protection of public health in the Beibu Gulf region and the mitigation of HAB events.

## 1. Introduction

The genus *Alexandrium* is an important toxic and harmful algal blooms (HABs) dinoflagellate distributed in coastal waters worldwide [1]. This genus was first described by Halim [2] and it has since become one of the most studied dinoflagellate genera. Among the over 30 species of *Alexandrium* reported to date, at least half can cause paralytic shellfish poisoning (PSP) [3,4]. Paralytic shellfish toxins (PSTs) accumulate in shellfish, fish, and other organisms through the food web. Humans who consume shellfish containing PSTs can become poisoned; PSTs thus represents a major health hazard [1,5]. *Alexandrium* is a common species in the coastal waters of China. Over the past 15 years, 24 HABs due to *Alexandrium* spp. have occurred in the Bohai, Yellow, and South China Seas [5], with disastrous consequences for local marine fisheries, ecosystems, and coastal landscapes.

The morphological identification of *Alexandrium* is based primarily on the fine structure of the apical pore complex, the first apical and sixth precingular plates, the presence/absence of a ventral pore and its location, the shape and size of sulcal plates, and whether they form chains [6,7,8]. However, morphological differences between the many species of *Alexandrium* are small. Some characteristics are plastic, such as the presence or absence of a ventral pore and the position of the anterior connecting pore. The identification of *Alexandrium* is thus inadequate when only morphological standards are applied. One newer method of identifying marine microalgae is DNA molecular identification. Applying this method to *Alexandrium* classification along with phylogenetic studies can help to identify species in this genus [8,9]. Studies on the molecular identification of *Alexandrium* have mostly focused on its large (LSU rDNA) and small (SSU rDNA) subunit, and non-coding internal transcribed spacer (ITS) ribosomal DNA sequences. For example, the *Alexandrium tamarense* species complex can be classified into *A. catenella* (Group Ⅰ, conspecific as *A. fundyense*), *A. mediterraneum* (Group Ⅱ), *A. tamarense* (Group Ⅲ), *A. pacificum* (Group Ⅳ), and *A. australiense* (Group Ⅴ) based on rDNA sequences [6,10,11,12]. New species *A. diversaporum*, *A. fragae,* and *A. pohangense* have recently been identified based on their cell morphology and 18S rDNA, 28S rDNA, and ITS ribosomal gene sequences [8,13,14]. Gu et al. [15,16] also isolated and germinated multiple strains of *Alexandrium* from cysts in sediments along the coast of China, and discovered the newly recorded species, *A. andersonii*, *A. fraterculum*, *A. leei*, *A. ostenfeldii*, *A. pseudogonyaulax*, *A. tamutum* through LSU rDNA and ITS sequence analysis.

Beibu Gulf is a natural, semi-enclosed, shallow gulf located in the northwestern continental shelf of the South China Sea. It is bordered on the west, north, and east by Vietnam, Guangxi, and Hainan Island, respectively. Beibu Gulf has long been regarded as the last clean sea zone in Chinese coastal waters. However, the number, scale, and areas of algal blooms in the region have significantly increased recently, and the number of species causing HABs has also increased [17,18]. Accurate identification of causative species is the biological basis for HAB prevention and monitoring. For *Alexandrium* spp. in the Beibu Gulf, a detailed investigation is needed. *Alexandrium* cysts in sediments from the Beibu Gulf near Fangchenggang have been germinated and then identified by optical microscopy, scanning electron microscopy, and LSU rDNA and ITS sequence analyses as *A. affine*, *A. andersonii*, *A. leei*, *A. pseudogonyaulax*, and *A. pacificum* (as Atama complex, Group IV) [6,10,16]. Cysts in samples from Fangchenggang and Beihai have been identified by optical microscopy as *A. minutum* and *A. pacificum* (as *A. tamarense*) [6,10,19,20]. *Alexandrium pacificum* (as *A. tamarense* and *A. catenella*) [6,10,11,21,22] is so far the only species that have been recorded from the waters in Beibu Gulf. Although *Alexandrium* cysts serve as seed banks in algal blooms, the species composition and toxicity of *Alexandrium* in the waters are inconsistent with these cysts [23,24]. Therefore, the species composition of the natural waters of Beibu Gulf needs further study.

*Alexandrium catenella*, *A. minutum*, *A. ostenfeldii,* and *A. pacificum* (as *A. tamarense*) species complex from the Bohai, Yellow, East China Sea, and South China Seas contain PSTs [6,10,16,25,26]. Wang et al. [27] also detected C1, C2, gonyautoxins (GTX)3, and 5, and NEO in the ATGX02 strain of *A. pacificum* (as *A. tamarense*) germinated near Weizhou Island in the Beibu Gulf, among which, C2 and GTX5 accounted for the highest proportion (>90%). The toxin content was 30.67–40.97 fmol/cell, but the PSP toxin was undetectable in the AAGX01 strain of *A. affine*. Zou et al. [26] tested the same ATGX02 strain of *A. pacificum* and found a toxin that included GTX1, 2, 3, and 4, dcSTX, and STX, which differed from that found by Wang et al. [27]. The toxin content was 4.03 fmol/cell, and the toxicity was 0.69 pg STXeq/cell [26], classifying it as a low-toxicity algal strain. In the study of Gu et al. [16], a total of 18 strains of *A. affine*, *A. andersonii*, *A. leei*, *A. pseudogonyaulax*, and *A. pacificum* (as Atama complex, Group IV) were germinated from cysts in the Beibu Gulf near Fangchenggang, but these strains’ PSTs were not analyzed. Currently, data about toxin production by *Alexandrium* strains in the natural waters of the Beibu Gulf remain scant, although PST contamination of shellfish was reported in this area [28,29,30], and occasionally exceeds food safety thresholds [28,29]. The source of those PSTs remains unknown.

Here we classify the species and define the toxicity of *Alexandrium* isolates from Beibu Gulf waters and characterize the toxin-producing algae that can cause PST contamination of shellfish. We isolated eight *Alexandrium* strains from Weizhou Island in the Beibu Gulf, an area with a high incidence of HABs [17] and analyzed their characteristic LSU rDNA, SSU rDNA, and ITS sequences to achieve their species identification. We also applied liquid chromatography-tandem mass spectrometry (LC-MS/MS) to analyze toxin composition and content. Here, we describe the molecular phylogeny of three *Alexandrium* species in the Beibu Gulf, their relationships with *Alexandrium* in other marine areas, and differences in toxin production.

## 2. Results

### 2.1. Sequence Analysis

The average lengths of the LSU rDNA, SSU rDNA, and ITS sequences obtained from the eight strains of *Alexandrium* from the Beibu Gulf were 1382, 1712, and 586 bp, respectively. The average base composition of the LSU rDNA sequences was 27.0% A, 30.8% T, 25.5% G, and 16.7% C, respectively, and the A+T content was greater than the G+C content (57.8% vs. 42.2%). The average base composition of the SSU rDNA sequences was 27.6% A, 29.5% T, 25.1% G, and 17.8% C, and the A+T content was greater than G+C content (57.1% vs. 42.9%). The average base composition of the ITS sequences was 24.4% A, 33.4% T, 23.9% G, and 18.3% C, and the A+T content was greater than G+C content (57.8% vs. 42.2%). These sequence composition characteristics were similar to those of the *Alexandrium* sequences that were used to construct the phylogenetic tree (Table 1). Among the three sequences, the SSU rDNA sequence had the highest and lowest proportions of conserved (82.6%) and variable (15.5%) sites, respectively, confirming that the SSU rDNA sequence is more conserved than the LSU rDNA and the ITS sequences (Table 1).

### 2.2. Phylogenetic Tree Analysis

**Figure 1:**
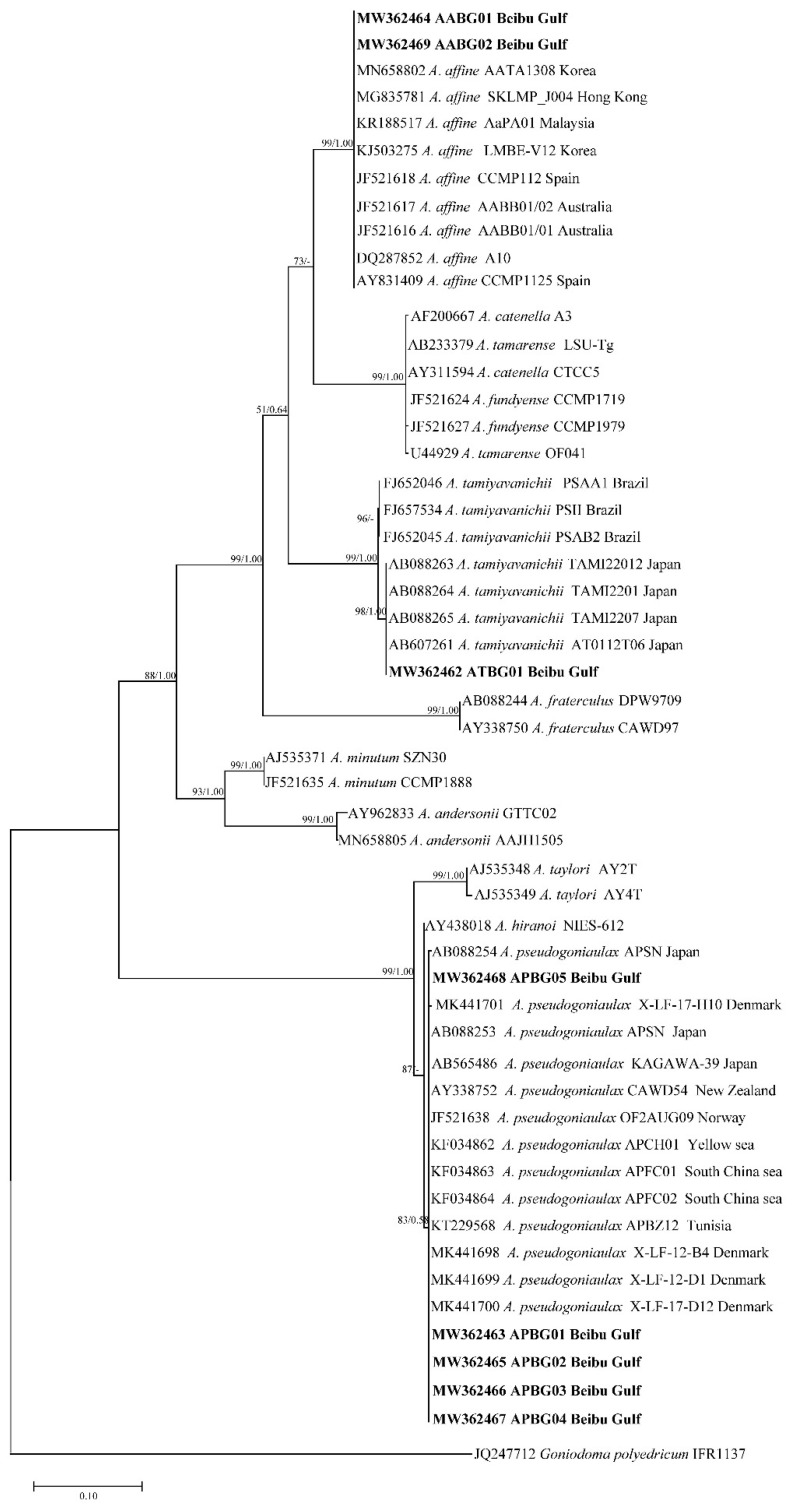
Phylogenetic tree constructed based on *Alexandrium* large subunit ribosomal DNA (LSU rDNA) sequences. Values at nodes indicate bootstrap values from the maximum likelihood method and posterior probabilities from the Bayesian inference method. Bootstrap values <50 and posterior probabilities <0.50 are not shown.

**Figure 2:**
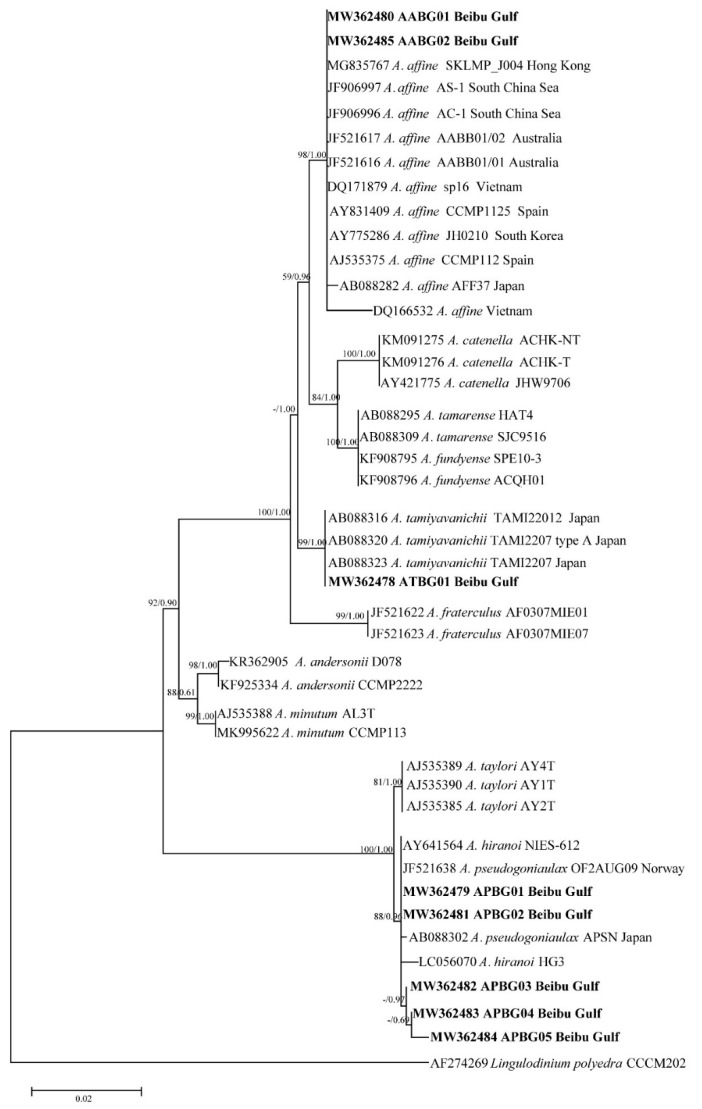
Phylogenetic tree constructed based on *Alexandrium* small subunit ribosomal DNA (SSU rDNA). Values at nodes indicate bootstrap values from the maximum likelihood method and posterior probabilities from the Bayesian inference method. Bootstrap values <50 and posterior probabilities <0.50 are not shown.

**Figure 3:**
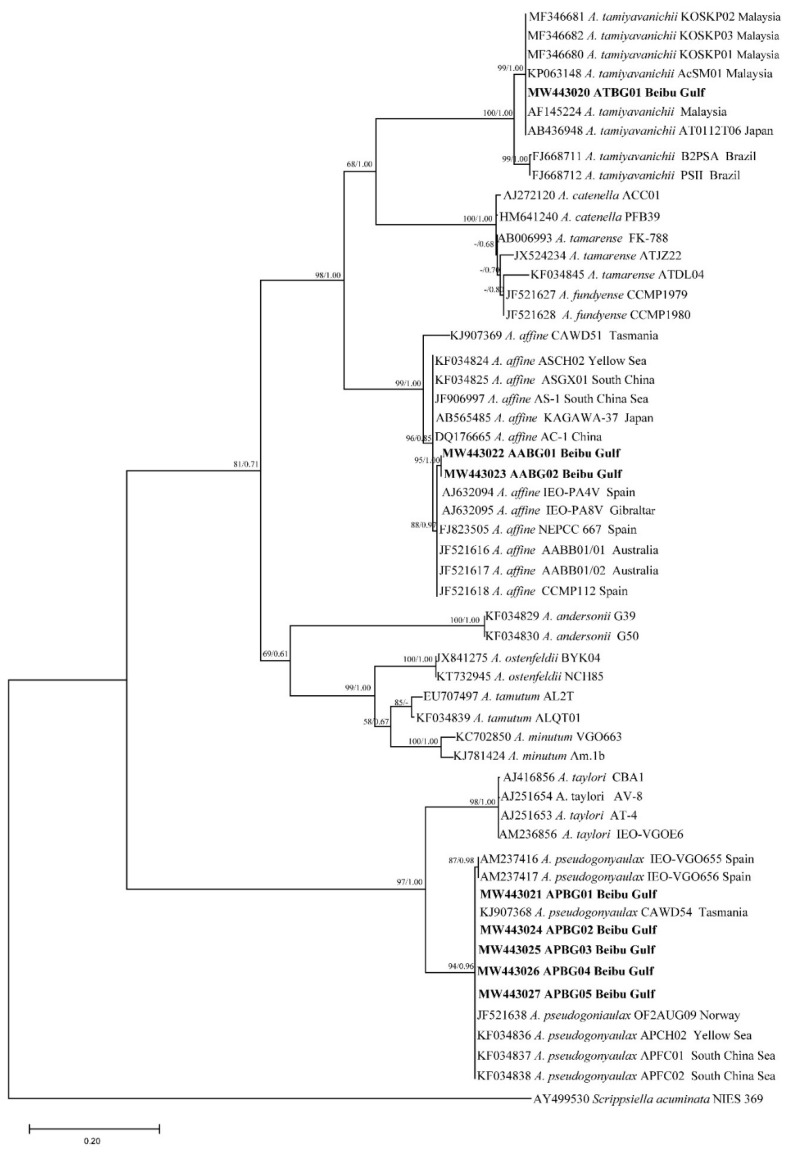
Phylogenetic tree constructed based on *Alexandrium* internal transcribed spacer (ITS) sequences. Values at nodes indicate bootstrap values from the maximum likelihood method and posterior probabilities from the Bayesian inference method. Bootstrap values <50 and posterior probabilities <0.50 are not shown.

Phylogenetic trees of the LSU rDNA, SSU rDNA, and ITS sequences were constructed using *Goniodoma polyedricum*, *Lingulodinium polyedra*, and *Scrippsiella acuminata* as outgroups (Figure 1, Figure 2 and Figure 3). The topological structures of the trees using the maximum likelihood and Bayesian inference were generally consistent; thus, only the maximum likelihood trees are shown. The three phylogenetic trees all showed that five of the *Alexandrium* strains from the Beibu Gulf (APBG01, APBG02, APBG03, APBG04, APBG05) and *A. pseudogonyaulax* from various marine regions worldwide clustered into a large branch, with bootstrap values/Bayesian posterior probabilities of 83/0.58, 88/0.96, and 94/0.96, respectively. The Beibu Gulf ATBG01 strain clustered with *A. tamiyavanichii* from other regions, with bootstrap values/Bayesian posterior probabilities of 99/1.00, 99/1.00, and 100/1.00, respectively. Two other *Alexandrium* strains (AABG01, AABG02) from Beibu Gulf clustered with *A. affine* from other marine regions, with bootstrap values/Bayesian posterior probabilities of 99/1.00, 98/1.00, and 99/1.00, respectively (Figure 1, Figure 2 and Figure 3). *A. tamarense*, *A. fundyense*, and *A. catenella* clustered together in a large branch, with bootstrap values/Bayesian posterior probabilities of 99/1.00, 84/1.00, and 100/1.00, respectively, and the remaining *Alexandrium* species also formed clear branches with strong bootstrap values and posterior probabilities (Figure 1, Figure 2 and Figure 3).

The Jukes–Cantor genetic distance matrix calculated based on LSU rDNA, SSU rDNA, and ITS sequences showed genetic distances among the *A. pseudogonyaulax* APBG01, APBG02, APBG03, APBG04, APBG05 strains of 0.000–0.014, 0.000–0.004, and 0.002–0.028, respectively. The *A. pseudogonyaulax* strains with the highest homology were the APFC01 and APFC02 strains from the South China Sea (LSU), the OF2AUG09 strain from Norway (SSU), the APFC01 and APFC02 strains from the South China Sea, the APCH01 strain from the Yellow Sea, and the CAWD54 strain from New Zealand (ITS), with genetic distances of 0.000–0.002, 0.000–0.004, and 0.000, respectively, whereas the genetic distances of LSU rDNA, SSU rDNA, and ITS from other *A. pseudogonyaulax* strains worldwide were 0.000–0.021, 0.000–0.009, and 0.000–0.037, respectively. The intraspecific genetic distances of the three sequences of *A. pseudogonyaulax* were 0.000–0.021, 0.000–0.009, and 0.000–0.037, respectively, and generally smaller than the interspecific genetic distances of 0.009–0.881, 0.000–0.111, and 0.206–1.368 for *Alexandrium* species.

The genetic distances of the LSU rDNA, SSU rDNA, and ITS sequences of the two *A. affine* AABG01, AABG02 strains from Beibu Gulf were 0.000, 0.000, and 0.005, respectively. They were most closely related to the Korean LMBE_V129 (LSU), Vietnamese sp16 (SSU), and Spanish IEO-PA4V (ITS) strains of *A. affine*, with genetic distances of 0.001, 0.000, and 0.006, respectively, whereas the genetic distances of LSU rDNA, SSU rDNA, and ITS with the other worldwide *A. affine* strains were 0.001–0.015, 0.000–0.008, and 0.006–0.072, respectively. The intraspecific genetic distances of the three *A. affine* sequences were 0.000–0.020, 0.000–0.009, and 0.000–0.072, respectively, and also significantly smaller than the interspecific genetic distances of 0.100–0.576, 0.011–0.089, and 0.319–1.219 from other *Alexandrium* species.

The ATBG01 strain of *A. tamiyavanichii* from Beibu Gulf was most closely related to the Japanese AT0112T06 (LSU) and TAMI22012 (SSU) strains and the Malaysian AcSM01 (ITS) strain, with genetic distances of 0.000, 0.001, and 0.000, respectively, whereas the genetic distances of LSU rDNA, SSU rDNA, and ITS with other *A. tamiyavanichii* strains worldwide were 0.000–0.013, 0.001–0.002, and 0.000–0.060, respectively. The intraspecific genetic distances of the three *A. tamiyavanichii* sequences were 0.000–0.013, 0.000–0.002, and 0.000–0.060, which were also significantly smaller than the interspecific genetic distances of 0.138–0.881, 0.011–0.097, and 0.357–1.368 from other *Alexandrium* species.

### 2.3. Toxin Analysis

Table 2 shows the toxins analyzed in the Beibu Gulf APBG04 strain of *A. pseudogonyaulax* and the ATBG01 strains of *A. tamiyavanichii*, as well as the PST toxin composition and content. No PSTs were detectable in *A. pseudogonyaulax* APBG04, whereas multiple PST congeners were detected in *A. tamiyavanichii* ATBG01, including GTX1,4, GTX2,3, GTX5, and STX, at 0.88, 1.2, 0.32, and 2.20 fmol/cell, respectively (mole percent: 19.1%, 26.1%, 7.0%, and 47.8%, respectively); the total PST content was 4.60 fmol/cell.

## 3. Discussion

*Alexandrium* species are distributed worldwide, and many of them are toxic [1]. Determining the species composition, toxicity, and biogeographic distribution of *Alexandrium* spp. in a marine area is a prerequisite for HAB control and PSP mitigation. *Alexandrium margalefii* and *A. tamiyavanichii* are recently discovered species in China using optical and fluorescence microscopy [31,32]. Ten species of *Alexandrium*, *A. affine*, *A. andersonii*, *A. catenella*, *A. fraterculum*, *A. leei*, *A. minutum*, *A. ostenfeldii*, *A. pseudogonyaulax*, *A. pacificum* (as *A. tamarense*), and *A. tamutum*, have been identified in the East China, South China, Yellow, and Bohai Seas using optical, fluorescence and scanning electron microscopy, and rDNA molecular identification techniques [6,10,16,33]. While *A. acatenella*, *A. catenella* (as *A. fundyense*), *A. hiranoi*, *A. minutum*, *A. ostenfeldii*, *A. pseudogonyaulax*, *A. pacificum*, and *A. tamiyavanichii* were identified in Hong Kong waters morphologically [6,34], but molecular sequences are not available to confirm their identities. Considering the identification of *Alexandrium* is inadequate when only morphological standards are used, the real number of *Alexandrium* species in Chinese waters still remains to be determined.

Eight Beibu Gulf *Alexandrium* strains were identified based on LSU rDNA, SSU rDNA, and ITS sequences as *A. affine, A. pseudogonyaulax*, and *A. tamiyavanichii*. These results are unsurprising, as *A. pseudogonyaulax* and *A. affine* are globally distributed and have been reported in the sediments of Beibu Gulf previously [16], whereas *A. tamiyavanichii* is a warm-water species distributed in temperate to tropical regions such as Japan, Thailand, Malaysia, Brazil, and Mexico [35,36,37,38,39]. Both *A. affine* and *A. pseudogonyaulax* have been identified in the East China, South China, and Yellow Seas [16], while *A. tamiyavanichii* has been found only in the East China Sea [31] and in waters near Hong Kong [34] based on morphology. This is the first report of *A. tamiyavanichii* from the Beibu Gulf confirmed with molecular sequences. This is also the first report on the toxicity of *A. tamiyavanichii* from Chinese coastal waters.

Except for the *A. tamarense* species complex, the three phylogenetic trees generally formed clear branches of *Alexandrium* species with good bootstrap values. The positions of the three Beibu Gulf species on the trees were consistent with those of other published studies [13,14], indicating that *Alexandrium* species can be distinguished using LSU rDNA, SSU rDNA, and ITS sequences. However, the phylogenetic tree constructed using SSU rDNA has occasional exceptions, because *A. hiranoi*, *A. pseudogonyaulax*, and strains APBG01–05 clustered together (Figure 2). *Alexandrium hiranoi* and *A. pseudogonyaulax* are difficult to identify using SSU rDNA gene sequences, which have been noted previously [13,40].

Generally, algal strains of the same species from the same or similar geographic locations have small base differences and are closely related; the present findings on *A. tamiyavanichii* are consistent with this. *A. tamiyavanichii* from the Beibu Gulf is most closely related to the Japanese strains AT0112T06 and TAMI22012, and the Malaysian strain AcSM01, and relatively distantly related to the Brazilian PSII strain. A relationship between the different strains of *A. affine* and *A. pseudogonyaulax* in terms of genetic distance and geographical was not obvious, however. For example, in the ITS tree, Beibu Gulf *A. affine* had close homology with strains of this species from the geographically distant waters of Spain, Australia, and Gibraltar, and less homology with algal strains from the geographically close waters of China and Japan. The LSU rDNA and ITS trees of *A. pseudogonyaulax* also showed that strains with different geographical origins clustered on the same large branch with no obvious small-branch topology. Others have also noted similar phenomena [14,16], which might be associated with the physiological and ecological features of *Alexandrium* species [1,4,41].

Previous tests of *A. pseudogonyaulax* did not reveal any PSTs, but did find the toxin goniodomin A (GDA) [40,42,43]. We did not detect PSTs in five strains of Beibu Gulf *A. pseudogonyaulax* either; whether they can produce GDA awaits further investigation. Two strains of Beibu Gulf *A. affine* were lost during culture maintenance, so PSTs could not be analyzed. Current literature suggests that *A. affine* is largely nontoxic [16,44] or only potentially toxic [45]. However, *A. affine* strains with low PST toxin content (<1.0–2.28 fmol/cell), including NeoSTX, STX, and GTX1-4, have been isolated from Ha Long Bay, Vietnam, near the Beibu Gulf [46]. 

*A. tamiyavanichii* strains from various marine areas worldwide, including the Beibu Gulf strain described herein, can produce PST toxins, but toxin composition and content differ significantly (Table 3). 

We detected only GTX1-5 and STX toxins in the Beibu Gulf strain, which is similar to the PSTs composition of the Chula 6 strains from Thailand and the KOSKP01, KOSKP02, and KOSKP03 strains from Malaysia [38,47,57], but different from other *A. tamiyavanichii* strains from these countries as well as strains from Japan, Brazil, and South Africa (Table 3). The range in PST content of *A. tamiyavanichii* reported in the literature is 1.17 fmol/cell–3.07 × 10^6^ fmol/cell. The PST content of one *A. tamiyavanichii* strain isolated from the recent cases of PSP outbreak at Kuantan Port, Malaysia [38] reached an extreme maximum of 3.07 × 10^6^ fmol/cell, which is 100,000 times higher than strain ATBG01 in this study. The Beibu Gulf *A. tamiyavanichii* strain is a low-toxicity algal strain, but more strains need to be tested in the future, and measurements from bloom populations are needed as well. 

Based on this and previous studies, Beibu Gulf *Alexandrium* species appear to be either non-toxic or weakly toxic [16,26,27]. This is, however, inconsistent with the high level of PST detection rate in Beibu Gulf shellfish, which have exceeded food safety standards on multiple occasions (Table 4). 

Possible explanations for this inconsistency include the following:

(1) Low-toxicity algal strains are widely distributed in the Beibu Gulf and survive for long periods. PSTs can thus accumulate in shellfish through time to levels sufficient to exceed regulatory standards. Anderson et al. [62] noted a similar phenomenon in Daya Bay, China where *A. pacificum* has very low toxicity of 7.2–12.7 fmol/cell, yet PSP poisoning still occasionally occurs there.
(2) Based on reports of *A. tamiyavanichii* toxin content varying by more than 5 orders of magnitude among isolates from the same locations in Malaysia and by factors of 857 in Thailand and 651 in Japan (Table 3), it seems very likely that other highly toxic *A. tamiyavanichii* strains occur in the Beibu Gulf that were not isolated. Low-abundance *Alexandrium* spp. in seawater can be missed due to limitations of small numbers of culture isolations, and thus highly toxic strains could easily be overlooked. Furthermore, because isolates were only established from Weizhou Island in the Beibu Gulf, and not from other highly productive areas of the Gulf, such as Qinzhou Bay and Tieshan Harbor, the presence of highly toxic species in these regions cannot be ruled out. In this regard, it is of note that the PST composition of shellfish from the Beibu Gulf differs significantly from the *A. tamiyavanichii* isolates analyzed here (Table 3 and Table 4). This could reflect biotransformation (see below) and/or different toxic *Alexandrium* species or strains from those analyzed here.
(3) *Alexandrium* in field populations can grow faster and produce more toxins than laboratory cultures, as has been observed by others through variations in temperature, salinity, light, and nutrients [63,64,65,66]. Brosnahan et al. [67] recently reported in situ growth rates for *A. catenella* that were more than twice those observed in laboratory cultures under similar growth conditions.
(4) Metabolism and bioconversion processes in different shellfish greatly affect the accumulation and potency of PSTs [68,69]. For example, the PSP toxicity of *Paphia undulata* is over 1000 times higher than that of *Meretrix lusoria* in the Beibu Gulf [29]. Thus, further study of biotransformation of PSTs in different shellfish from the Beibu Gulf is needed.
(5) Although *Alexandrium* spp.are the principal causative organisms of PSP in many regions, the presence of other PST-producing microalgae, such as *Gymnodinium catenatum* and *Pyrodinium bahamense* [70,71], and atypical toxin-producing organisms such as brackish cyanobacteria, as well as calcareous red macroalgae [72], cannot be ruled out.

## 4. Conclusions

The Beibu Gulf region is experiencing rapid economic growth, frequent HABs, and the increasingly serious problem of PST contamination of shellfish. To explore this issue, potential PST toxin-producing algae in the *Alexandrium* genus were isolated from Weizhou Island, a HAB hotspot in the Beibu Gulf and characterized phylogenetically and toxicologically. For the first time, *A. affine*, *A. pseudogonyaulax*, and *A. tamiyavanichii* were definitively identified in plankton samples from the Beibu Gulf, among which, *A. tamiyavanichii* is new to this body of water. This species showed small base differences from Asian strains of the same species from nearby locations. There was no observed relationship between genetic distance and the geographical distribution of various *A. affine* and *A. pseudogonyaulax* strains. *A. pseudogonyaulax* strains did not produce PSTs, whereas *A. tamiyavanichii* produced 4.6 fmol/cell of toxin, indicating that it is a low-toxicity strain in comparison to global isolates. This finding of low toxicity is inconsistent with the detection of relatively high levels of PSTs in shellfish from the Beibu Gulf, suggesting that higher toxicity strains may occur in those waters, but were unsampled. Other explanations including biotransformation of PSTs in shellfish and the presence of other PST-producing algae are also suggested. This is the first report of toxic *A. tamiyavanichii* in the coastal waters in China. In future studies, potential PST-producing microalgae and toxic shellfish should be collected from the major localities in the Beibu Gulf to further elucidate species diversity, biogeographical distribution, toxin-producing characteristics, and relationships with PST vectors in the region. This would provide much-needed insights into PST sources and aid in the protection of public health in the Beibu Gulf.

## 5. Materials and Methods

### 5.1. Algal Source and Culture Conditions

**Figure 4:**
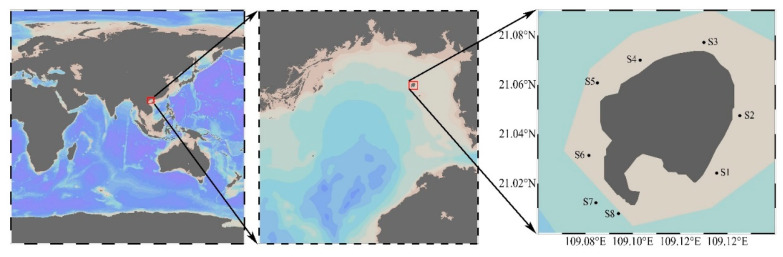
Sampling location.

Water samples containing phytoplankton collected from the waters of Weizhou Island in the Beibu Gulf four times between the summer 2018 and summer 2019 (Figure 4), were transported and stored as described in the “Specification for Marine Monitoring” (GB/T17378-2007). The samples were visually assessed using a TS100 inverted microscope (Nikon Corp., Tokyo, Japan). Algal cells of interest were isolated from water samples using capillary tubes, washed 3 or 4 times with sterilized seawater, then cultured, resulting in eight *Alexandrium* strains (Table 5). The culture conditions were f/2 medium, 22 °C, light intensity 150 μE/m^2^/s, and a 12:12 h light-dark cycle. The algal strains ATBG01 and APBG0 were cultured to the exponential growth phase, then toxin was extracted from 6 × 10^6^ algal cells.

### 5.2. Extraction of DNA, PCR Amplification, and Sequencing 

Fresh algae (30 mL) in the exponential growth phase were collected and sedimented by centrifugation at 7000× *g* for 1 min, then the supernatants were discarded. Total DNA was obtained using BioFastSpin Plant Genomic DNA Extraction Kits (Bioer, Hangzhou, China), and characteristic LSU rDNA, SSU rDNA, and ITS fragments were amplified by PCR. The amplification primers (5′→3′) were listed in Table 6. The PCR reaction volume was 40 µL, and included 20 µL of 2 × Es Taq Master Mix (CoWin Biosciences, Cambridge, MA, USA), 1 µL of DNA template, 17 µL of ddH_2_O, and 1 µL each of forward and reverse primer. The PCR program comprised pre-denaturation at 94 °C for 5 min, 35 cycles of 94°C for 30 s, 56 °C for 30 s, and 72 °C for 30 s, and elongation at 72 °C for 5 min. The PCR products were sequenced at a commercial laboratory (TsingKe Biological Technology Company (Beijing, China).

### 5.3. Sequence Analyses

We searched the LSU rDNA, SSU rDNA, and ITS sequences of genes from the eight *Alexandrium* strains in the NCBI database using BLAST, and downloaded 43 LSU rDNA, 34 SSU rDNA, and 44 ITS sequences with high species similarity and coverage. We used the online BioEdit v7.2.5 software (https://bioedit.software.informer.com/, accessed on 8 January 2021), for multiple sequence alignment of the obtained sequences. We calculated and analyzed genetic distances within and between species and calculated base compositions, conserved, variable, parsimony informative, and singleton sites, and transition/transversion ratios using online MEGA-X v10.1.5 software (https://www.megasoftware.net/, accessed on 10 January 2021). We confirmed that GIR+I+G was the best alternative model using the AIC function in downloadable Modeltest3.7 software (https://sourceforge.net/projects/modeltest/, accessed on 10 January 2021). We constructed a maximum likelihood phylogenetic tree for the sequences obtained using MEGA-X v10.1.5 software, selected GIR+I+G as the best alternative model, and tested the confidence of the branches by running 1000 bootstrap replications. We constructed a Bayesian phylogenetic tree using Bayesian inference (BI) in MrBayes v3.1.2 software (http://nbisweden.github.io/MrBayes/, accessed on 13 January 2021), again with GIR+I+G as the best replacement model, and 4 Markov chain operations were run for 1 × 10^6^ generations for Bayesian analysis, with sampling every 500 generations. The phylogenetic tree was viewed and downloaded using FigTree v1.4.0 software, and edited using Adobe Acrobat DC v2020.013.20074.

### 5.4. Toxin Extraction

Algal suspensions (6 L) in the exponential growth phase were centrifuged at 4000 rpm for 8 min (15 °C), the supernatant was decanted, and the sedimented microalgal cells were stored at −80 °C. After three freeze-thaw cycles, stored algal cells were suspended in 10 mL of 0.1 mol/L acetic acid, sonicated on ice for 20 min, and centrifuged at 8000 rpm for 10 min at 4 °C. The supernatant was collected, the pellet was extracted again, the supernatants were combined and adjusted to a volume of 20 mL with 0.1 mol/L acetic acid, and 1.0 mL was passed through 0.22 µm mobile phase filter membranes (Shanghai ANPEL Laboratory Technologies Inc., Shanghai, China) for LC-MS/MS analysis.

### 5.5. Liquid Chromatography-Mass Spectrometry 

We analyzed the PSP toxins, GTX1, GTX2, GTX3, GTX4, GTX5, dcGTX2, dcGTX3, STX, dcSTX, neoSTX, dcNEO, C1, and C2 using liquid chromatography-tandem mass spectrometry (LC-MS/MS). Chromatographic conditions: These comprised a Welch Ultimate Amide chromatographic column (4.6 × 150 mm × 3.5 μm); mobile phase A, 2 mmol/L ammonium formate (containing 50 mM formic acid); B, acetonitrile; injection volume, 10 μL; column temperature, 40 °C; flow rate, 0.6 mL/min. The gradient elution program was 0–1 min, 90% A; 1–3 min, linear gradient from 90% A to 10% A; 3–10 min, 10% A; 10–11 min, linear gradient from 10% A to 90%; 11–13 min, 90% A.

Mass spectrometry conditions: ESI ion source, positive/negative polarity switching scan mode, capillary voltage, 4000 V; nozzle voltage, 500 V; sheath gas temperature, 350 °C; sheath gas flow, 11 L/min; drying gas temperature, 320 °C; drying gas flow, 5 L/min; nebulizer pressure, 45 psi. Specific multiple reaction monitoring parameters and conditions are shown in Table 7.

## Figures and Tables

**Table 1:** Analysis of *Alexandrium* gene sequences used in a phylogenetic tree.

Gene	Analysis Length	Average Content (%)				Conserved Site	Variable Site	Parsimonious Information Site	Monomorphic Site	Conversion/Transversion Ratio
		A	T	G	C					
LSU rDNA	996	26.9	31.5	25.6	16.0	416	555	373	182	1.0
SSU rDNA	1769	27.7	29.4	25.2	17.7	1462	275	245	29	1.9
ITS	666	24.7	34.0	23.9	17.4	160	494	449	45	0.7

**Table 2:** The components and content of paralytic shellfish toxins (PSTs) in *Alexandrium* spp. from the Beibu Gulf (fmol/cell).

Toxins	*A. tamiyavanichii*	*A. pseudogonyaulax*
	ATBG01	APBG04
GTX1, 4	0.88	ND
GTX2, 3	1.2	ND
GTX5	0.32	ND
dcGTX2	ND	ND
dcGTX3	ND	ND
STX	2.20	ND
dcSTX	ND	ND
neoSTX	ND	ND
dcNEO	ND	ND
C1	ND	ND
C2	ND	ND
Total PST content	4.60	ND

**Table 3:** Paralytic shellfish toxins composition and contents in *Alexandrium tamiyavanichii*.

Strain	Locality	Toxin Component	Toxin Content (fmol/cell)	References
Chula 5	Gulf of Thailand	C1-2, GTX1-5, STX	16,200 ^a^	[47]
Chula 6	Gulf of Thailand	GTX1-5, STX	7500 ^a^	[47]
Chula 8	Gulf of Thailand	C1-3, GTX1-5, STX	18.9	[48]
MBS8811-1	Sagami Bay, Japan	C1-4, GTX1-5, STX	3.7	[48]
MBS8811-3	Sagami Bay, Japan	C1-2, GTX1, 4, GTX5, STX	66.3	[48]
—	Seto Island, Japan	C1-2, GTX1-5, STX	112.5	[35]
—	Malaysia	—	26	[49]
CTCC23	South Africa	C1-4, GTX1-4, STX, neoSTX, B1	0.26 ^b^	[50]
Western Japan	Japan	C1-2, GTX1-5, STX, neoSTX	40–424	[51]
AcMS01	Sebatu Malacca, Malaysia	C1-2, GTX1-5, STX	38–80	[52]
AcMS01	Sebatu Malacca, Malaysia	C1-2, GTX1-5, STX	60–180	[53]
—	Malaysia	C2, GTX1-4, dcGTX3, STX	54	[54]
ATY041106	Seto Island, Japan	C1-2, GTX1-4	38.7 ± 10.9 × 10^−6 a^	[55]
ATY051018	Seto Island, Japan	—	111.5 × 10^−6 a^	[55]
Fukuyama Bay	Seto Island, Japan	C1-4, GTX1-5, neoSTX, STX	244 ± 102	[56]
Kasato Bay	Seto Island, Japan	C1-4, GTX1-5, neoSTX, STX	307 ± 83.8	[56]
Uchinoumi	Seto Island, Japan	C1-4, GTX1-5, neoSTX, STX	328 ± 152	[56]
Inokushi Bay	Kyushu Island, Japan	C1-4, GTX1-5, neoSTX, STX	54.7 ± 5.32	[56]
PSAA1	Brazil	GTX3-4, dcGTX2-3, neoSTX, STX	16.85	[36]
At2	Seto Island, Japan	C1-2, GTX1-6, neoSTX, STX	2410	[37]
At4	Seto Island, Japan	C1-2, GTX1-6, neoSTX, STX	840	[37]
At6-C1	Seto Island, Japan	C1-2, GTX1-6, neoSTX, STX	289	[37]
At6-C2	Seto Island, Japan	C1-2, GTX1-6, neoSTX, STX	359	[37]
At6-C3	Seto Island, Japan	C1-2, GTX1-6, neoSTX, STX	264	[37]
At6-C4	Seto Island, Japan	C1-2, GTX1-6, neoSTX, STX	220	[37]
KOSKP01	Kuantan Port, Malaysia	GTX1-5	3070	[57]
KOSKP02	Kuantan Port, Malaysia	GTX1-5	5960.4	[57]
KOSKP03	Kuantan Port, Malaysia	GTX1-5	1027.2	[57]
—	Kuantan Port, Malaysia	GTX1-5	3.07 × 10^6^	[38]
—	Sebatu Malacca, Malaysia	GTX1-5	1.167	[38]
ATBG01	Weizhou Island, Beibu Gulf	GTX1-5, STX	4.6	This study

^a^ MU/cell; ^b^ equivalent of Saxitoxin.

**Table 4:** PST contamination in shellfish in the Beibu Gulf, China.

Sampling Date	Locality	Methodology	Toxins	Detection Rate/Exceedance Rate	References
2001.11–2004.04	Tieshan, Beihai, Fangcheng, Weizhou	MBA, HPLC	C1-2, GTX1-5, STX	exceeding standard 2.15–3.54 times	[58]
2003.03–2004.05	Beihai	MBA, HPLC	C1-2, GTX1-4, STX	detection rate 8.30%	[59]
2005–2009	Beibu Gulf	MBA	—	exceedance rate 1.0%	[28]
—	Qinzhou, Fangcheng, Beihai	American ABRAXIS kit	—	— ^a^	[60]
2007.11–2008.10	Beihai	MBA	—	— ^b^	[61]
2014.09–2014.11	Weizhou, Beihai, Fangcheng, Qinzhou	HPLC	GTX1-5, dcGTX2-3, neoSTX, dcSTX, STX	detection rate 100%, exceedance rate 6.1%	[29]
2015.09	Qinzhou	MBA, HPLC	GTX1, GTX4, neoSTX, STX	detection rate 86%, no exceedance	[30]

MBA, Mouse Bioassay; HPLC, High Performance Liquid Chromatography; ^a^ positive results; ^b^ no PSP was found.

**Table 5:** Sampling information.

Strains	Collection Date	Location
ATBG01	2018-06-09	S2 (21°02′51″ N, 109°08′43″ E)
APBG01	2018-09-15	S2 (21°02′51″ N, 109°08′43″ E)
AABG01	2018-09-15	S2 (21°02′51″ N, 109°08′43″ E)
AABG02	2018-12-20	S8 (21°00′28″ N, 109°05′38″ E)
APBG02	2019-06-10	S2 (21°02′51″ N, 109°08′43″ E)
APBG03	2019-06-10	S4 (21°04′12″ N, 109°06′11″ E)
APBG04	2019-06-10	S5 (21°03′39″ N, 109°05′06″ E)
APBG05	2019-06-10	S5 (21°03′39″ N, 109°05′06″ E)

**Table 6:** Primers information.

Gene	Primers	Primer Sequences	References
LSU rDNA	D1R	5′-ACCCGCTGAATTTAAGCATA-3′	[73]
	D2C	5′-TGATCCTTCTGCAGGTTCACCTAC-3′	
SSU rDNA	1F	5′-AACCTGGTTGATCCTGCCAGT-3′	[74]
	1528R	5′-TGATCCTTCYGCAGGTTCAC-3′’	
ITS	FA	5′-CCAAGCTTCTAGATCGTAACAAGG(ACT)TCCGTAGGT-3′	[75]
	RB	5′-CCTGCAGTCGACA(TG)ATGCTTAA(AG)TTCAGC(AG)GG-3′	

**Table 7:** Multiple reaction monitoring conditions.

Toxins	Quantitative Transition *m*/*z*	Qualitative Transition *m*/*z*	Residence Time (ms)	Impact Voltage (V)	Fragmentor Voltage (V)
GTX1	412 > 332.1	412 > 314.2	100	6/20	100/100
GTX2	396.1 > 316.1	396.1 > 297.8	100	5/15	110/110
GTX3	396.1 > 316.1	396.1 > 298.7	100	5/15	110/110
GTX4	412 > 332.1	412 > 314.2	100	6/20	100/100
GTX5	380.1 > 300	380.1 > 282.1	100	11/15	94/105
dcGTX2	352.8 > 334.7	352.8 > 254.9	100	5/10	120/120
dcGTX3	352.8 > 334.7	352.8 > 254.9	100	5/10	120/120
STX	300 > 282	300 > 204	100	10/20	100/100
dcSTX	257 > 222.1	257 > 126	100	10/20	80/80
neoSTX	316.1 > 298	316.1 > 220	100	10/20	100/100
dcNEO	273 > 225.1	273 > 126	100	18/18	80/80
C1	474.1 > 251	474.1 > 122	100	20/30	60/60
C2	474.1 > 351	474.1 > 122	100	20/30	60/60

## Data Availability

Data are available upon request, please contact the correspondence author Yixiao Xu.
